# The two‐process model of sleep regulation: Beginnings and outlook^†^

**DOI:** 10.1111/jsr.13598

**Published:** 2022-05-03

**Authors:** Alexander Borbély

**Affiliations:** ^1^ Institute of Pharmacology and Toxicology University of Zurich Zurich Switzerland

**Keywords:** circadian pacemaker, circadian sleep–wake rhythm, forced desynchrony, homeostatic process, sleep regulation, two‐process model

## Abstract

The two‐process model serves as a major conceptual framework in sleep science. Although dating back more than four decades, it has not lost its relevance for research today. Retracing its origins, I describe how animal experiments aimed at exploring the oscillators driving the circadian sleep–wake rhythm led to the recognition of gradients of sleep states within the daily sleep period. Advances in signal analysis revealed that the level of slow‐wave activity in non‐rapid eye movement sleep electroencephalogram is high at the beginning of the 12‐light period and then declines. After sleep deprivation, the level of slow‐wave activity is enhanced. By scheduling recovery sleep to the animal's activity period, the conflict between the sleep–wake‐dependent and the circadian influence resulted in a two‐stage recovery pattern. These experiments provided the basis for the first version of the two‐process model. Sleep deprivation experiments in humans showed that the decline of slow‐wave activity during sleep is exponential. The two‐process model posits that a sleep–wake‐dependent homeostatic process (Process S) interacts with a process controlled by the circadian pacemaker (Process C). At present, homeostatic and circadian facets of sleep regulation are being investigated at the synaptic level as well as in the transcriptome and proteome domains. The notion of sleep has been extended from a global phenomenon to local representations, while the master circadian pacemaker has been supplemented by multiple peripheral oscillators. The original interpretation that the emergence of sleep may be viewed as an escape from the rigid control imposed by the circadian pacemaker is still upheld.

The two‐process model posits that sleep is regulated by interactions between a history‐dependent and a history‐independent process, and that the two processes can be monitored by empirical variables. That the number of citations per year of the original publication is still rising (Google Scholar Feb 2022) indicates its persistent relevance. In the following I shall retrace its origins and end by discussing some of its present applications.

## 1 ANIMAL EXPERIMENTS THAT LED TO THE MODEL

In 1976, Daan and Pittendrigh published a series of papers on the functional analysis of circadian pacemakers in rodents. Their impressive research was based on the rest–activity rhythm. The authors proposed a model in which two circadian oscillators mutually interact, and are coupled separately to sunrise and sunset (Pittendrigh & Daan, 1976). This two‐oscillator system could account for the behavioural adaptation to the seasonal variation of the photoperiod.

In the 1970s I conducted various studies on the effect of light on sleep and motor activity in the rat, and showed that short light–dark cycles exert selective effects on non‐rapid eye movement (NREM) sleep and rapid eye movement (REM) sleep (Borbély, 1975; for a review, see Borbély, 1978). Within the light phase, the rat's main sleep period, NREM sleep peaked in the early part, whereas REM sleep was highest in the late part. The question arose whether the two sleep stages are controlled by separate circadian oscillators related to the dark–light and light–dark transition. To gain further insights, we gradually extended the photoperiod from 12 to 20 hr by either phase‐advancing light‐onset or phase‐delaying dark‐onset (Borbély & Neuhaus, 1978a). Our electroencephalogram (EEG) telemetry system allowed us to record sleep concomitantly with motor activity and consummatory behaviour. The data confirmed that NREM sleep and REM sleep are partly independent processes, and indicated that REM sleep may be phase‐related to the light–dark transition. This study is significant because for the first time long‐term variations of animal sleep were investigated in the context of a circadian model.

A related study focused on the presence and absence of light (Borbély & Neuhaus, 1978b). Animals were recorded for several days under the following four schedules: light–dark 12:12 hr; continuous darkness; continuous light; and a skeleton photoperiod in which short light pulses were applied to maintain the synchrony of the circadian rhythm. These protocols, typical for circadian rhythm research, were applied to investigate sleep. We concluded that the circadian pattern of sleep and waking is largely due to intrinsic factors, and that the light–dark cycle has only a minor influence. Interestingly, the circadian amplitude of REM sleep and motor activity were consistently larger than those of NREM sleep and waking.

The key experiment leading to the formulation of the two‐process model was a sleep deprivation study in the rat (Borbély & Neuhaus, 1979). Its aim was to specify more closely the factors involved in sleep regulation. In human sleep, slow‐wave sleep (SWS) is a substage of NREM sleep where EEG slow waves predominate. SWS occurs preferentially in the first part of sleep. The question was whether a similar situation prevails in rodent sleep. To this end we submitted the EEG to an amplitude–frequency analysis where five frequency bands were defined.^1^ The lowest frequency bands representing slow waves showed a maximum at the beginning of the 12‐hr light phase, which was followed by a decline. This trend was reversed in the middle of the 12‐hr dark phase when a steep rise occurred. To assess the influence of prior waking, the animals were subjected to 12‐hr and 24‐hr sleep deprivation. In fact, the low‐frequency fraction of NREM sleep was increased in relation to the duration of the preceding waking period. A crucial part of the study was to make the 24‐hr sleep deprivation end at dark onset, the beginning of the animal's circadian activity period. This created a conflict between the increased sleep pressure and the circadian tendency for waking. The rise in low‐frequency activity occurred in two stages: a initial increase immediately following the enforced waking succeeded by a delayed increase 12 hr later.

In the discussion of these results, we argued that the circadian pacemaker schedules sleep and waking at predetermined phases of the 24‐hr cycle and thereby facilitates the adaptation of an animal to its environment. However, this rigid temporal schedule may prevent the adaptation of sleep and waking to the momentary needs. Added flexibility is conferred by the intensity dimension of NREM sleep. Thus, an increased sleep need can be fulfilled by intensifying sleep instead of prolonging sleep. This specific feature is limited to NREM sleep. A REM sleep deficit must be repaid in the hard currency of time. We considered sleep to be “gated” at the end of the activity phase when “sleep pressure” is released and becomes manifest by long NREM sleep episodes with a high proportion of slow waves. With these considerations, the discussion section of our paper (Borbély & Neuhaus, 1979) already contains the essence of the two‐process model.

## 2 FIRST VERSIONS OF THE MODEL

In 1979 I presented the first version of the model at a meeting on Functional States of the Brain that Martha Koukkou and Dietrich Lehmann organized in Sounion, Greece (Borbély, 1980). Based on our experimental data in the rat, I showed a figure in which the circadian rest–activity rhythm gates a recovery process that becomes manifest during the circadian rest phase. I proposed that the rest phase subserves forced immobility, while the sleep states ensure recovery. In the paper published in the congress proceedings, I coined the term “sleep homeostasis” to conceptualize the compensatory increase of SWS in relation to preceding waking.

**FIGURE 1:**
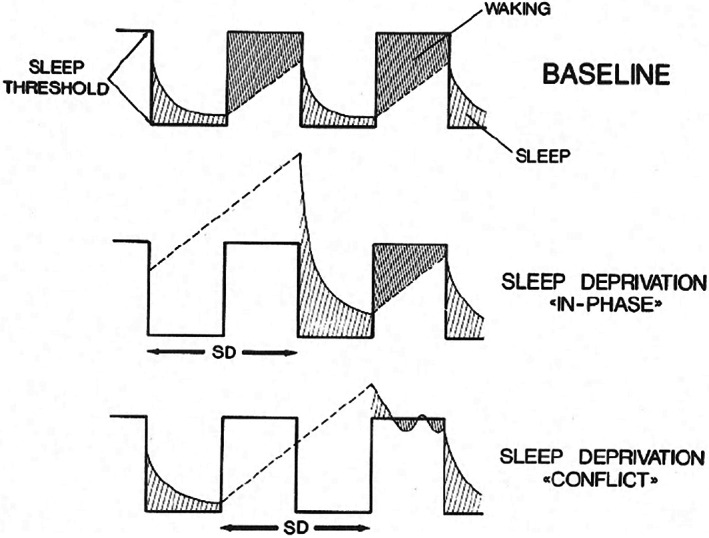
An early version of the two‐process model based on animal data. A circadian threshold gates a recovery process that increases during waking and declines exponentially during sleep. If recovery from sleep deprivation (SD) starts with the activity phase, a two‐stage rebound is observed (Borbély, 1982)

In 1980 at the International Congress of Physiology in Budapest, the title of my presentation referred for the first time explicitly to the two processes considered to underlie sleep^2^ (Borbély, 1981). My argument was that the sleep process is regulated relative to an internal reference level. In this paper I advanced various hypotheses of how sleep and recovery may be related (Figure 1).

## 3 VERSIONS OF THE MODEL BASED ON HUMAN DATA

In 1980 we used the sleep deprivation study in the rat as a template for a human experiment. Instead of the amplitude–frequency analysis, we applied a newly developed all‐night spectral analysis of the EEG based on an Fast Fourier Transform routine (Borbély et al., 1981). After an adaptation night and two consecutive baseline nights, the subjects remained awake for 40.5 hr and then slept on two consecutive recovery nights. The method of EEG signal analysis allowed a quantitative assessment of the changes in contrast to conventional sleep scoring. In addition, the data revealed trends across the frequency spectrum that were inadequately captured by the commonly used frequency bands. The most important result was that for both baseline sleep and recovery sleep the decline of EEG slow‐wave activity (SWA) over NREM–REM sleep cycles could be described by an exponential function with the same time constant. We concluded that SWA reflects a monotonically declining sleep process whose initial value is determined by the duration of prior waking.

In October 1980, I was invited to a meeting on the Vertebrate Circadian System organized by Jürgen Aschoff. Not being a rhythm scientist, I anticipated the event with some trepidation. Finally, I decided to present the early version of the two‐process model describing how the interaction of a sleep–wake‐dependent process with a circadian process accounts for the effects of sleep deprivation. Serge Daan, who had given the opening lecture, was galvanized by my presentation, because he realized that the model could explain internal desynchronization data with a single circadian pacemaker instead of two or more pacemakers as had been proposed in previous models.

Serge and I decided to join forces on the further development of the model. This was a fateful decision that initiated a close collaboration for almost 20 years paired with a lifelong friendship. Serge used the not yet published human sleep deprivation data to calculate the rate constants of the build‐up and breakdown of the sleep–wake‐dependent process. In his home lab in Groningen, he started a collaboration with Domien Beersma, a physicist working in the Psychiatric Clinic directed by Rutger van den Hoofdakker. In June 1981, the three of us attended a symposium on Mathematical Models of the Circadian Sleep–Wake Cycle organized by Martin Moore‐Ede and Charles Czeisler. It was a satellite meeting of a major sleep congress in which I gave a keynote lecture and presented for the first time the two‐process model based on human data.

In the model, human sleep regulation is described by the interaction of a sleep–wake‐dependent, homeostatic Process S (S for sleep in analogy with Pappenheimer's Factor S) and a circadian Process C (Borbély, 1982). The time course of S is based on SWA exhibiting an exponential decline in baseline sleep as well as in recovery sleep after sleep deprivation. A saturating exponential function, not yet based on empirical data, represents the rise in sleep pressure during waking. Process C is represented by a sine function whose phase position is derived from vigilance rhythm data during prolonged sleep deprivation. Total sleep propensity corresponds to the sum of S and C. In the model, the inverse of C represents the variation of the sleep threshold whose intersection with S defines the time of awakening. REM sleep is controlled by the circadian pacemaker and is inhibited by Process S. The model could simulate empirical data describing the variation of sleep duration as a function of sleep‐onset time. At that time, Serge Daan and Domien Beersma had already performed extensive quantitative simulations for a series of protocols that they reported in a book chapter scheduled to appear in 1982, which was unfortunately delayed until 1984.

In 1982, our Zurich team organized the congress of the European Sleep Research Society, which was preceded by a satellite symposium on models and followed by an informal workshop on related topics. The presence of foremost sleep scientists was an excellent occasion to present the merits of the modelling approach and to raise the interest of the sleep research community.

In 1982, Serge, Domien and I decided to publish a joint paper covering many facets of the model and to include quantitative simulations (Daan et al., 1984). Previously, my colleagues had added an upper threshold to the model that represented the time of sleep onset (Daan & Beersma, 1984). This threshold was incorporated in the new version of our model, where Process S as a “somnostat” oscillates between two thresholds. We assumed that external conditions modulate the level of the upper threshold. Sleep deprivation raises it, whereas bedrest, warmth, darkness and the absence of social stimuli lower it. By manipulating the threshold level and the amplitude of its modulation, internal desynchronization and circabidian patterns are obtained. Based on empirical data, a skewed sine wave was chosen for the circadian modulation. Empirical data obtained under temporal isolation and continuous bedrest were successfully simulated. We concluded that “a single circadian pacemaker, relatively insensitive to the internal milieu, would keep track of environmental time, while exerting gentle control over a homeostatic process that monitors internal demands.”

The paper was followed by a commentary of Richard Kronauer and Philippa Gander who defended their original two‐oscillator model (Kronauer et al., 1982) and argued that Process S could be also regarded as an oscillator. They pointed out that the resurgence of SWS at the end of extended sleep periods cannot be explained by Process S. In our reply we emphasized that the model could generate experimental designs to compare predictions and experimental results, and we conceded that a modification would be required if a systematic increase of slow waves at the end of sleep would be experimentally demonstrated. In the following years, we conducted a series of experiments to test the predictions of the model and also simulated the occasional resurgence of slow waves.

**FIGURE 2:**
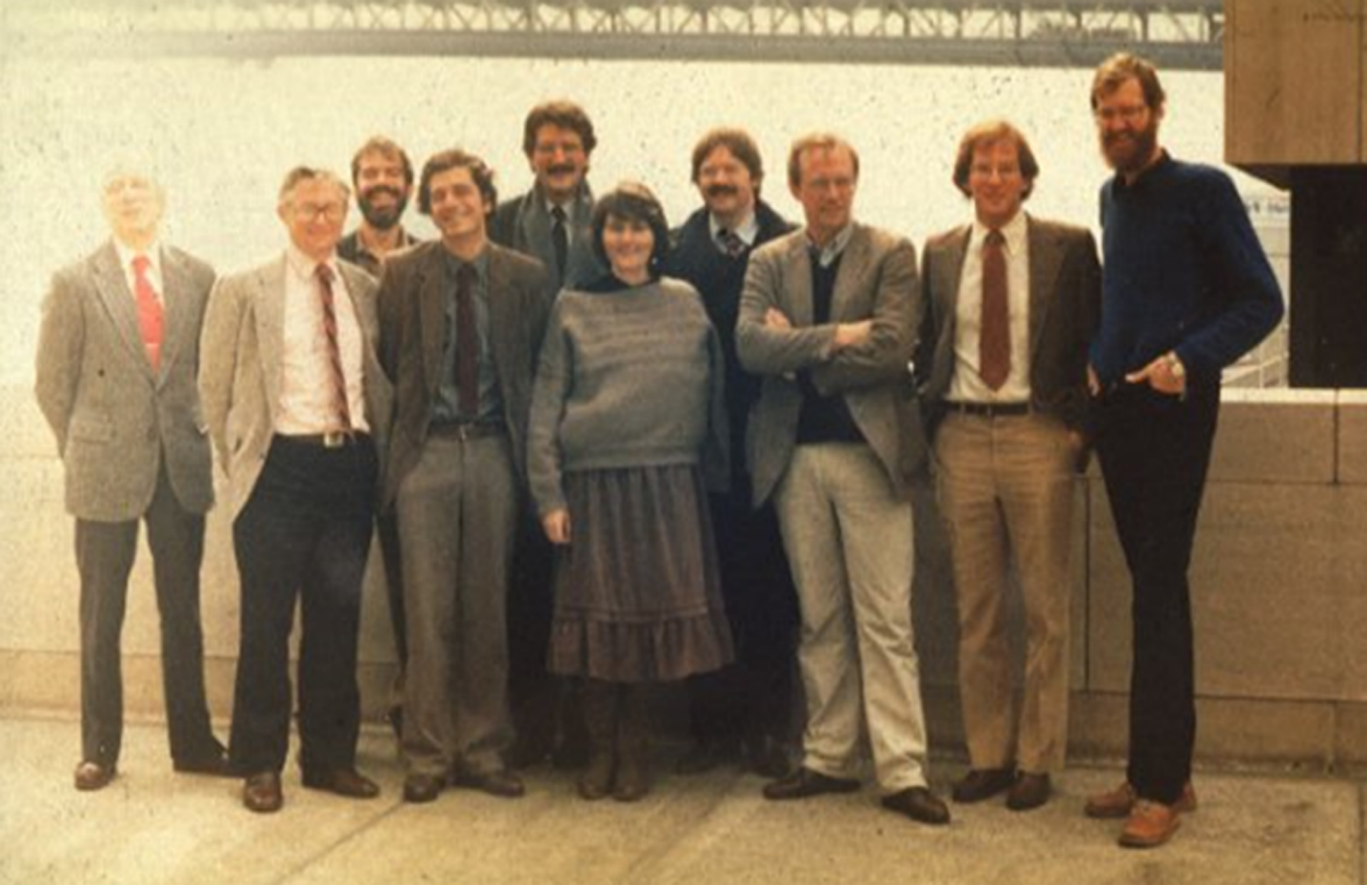
Participants of a meeting at Rockefeller University, New York in February 1985. Front row from left: Richard Kronauer, Chris Gillin, Alexander Borbély, Phillippa Gander, Serge Daan, Steven Strogatz, Domien Beersma. Back row from left: Michael Terman, Charles Czeisler, Tom Wehr

In 1985, I spent a sabbatical at the Neuroscience Institute in New York. There I had the possibility to organize, together with Serge Daan, a 2‐day meeting at Rockefeller University to discuss the merits of the two‐process model versus the two‐oscillator model. Richard Kronauer, Charles Czeisler, Steve Strogatz and Philippa Gander represented the Harvard group; Serge, Domien and I the Zurich‐Groningen group. Chris Gillin, Michael Terman and Tom Wehr were further knowledgeable participants. We had an intense discussion, though each group maintained its original position. One year later, the Harvard group still argued that at least in a mathematical sense our model employs two oscillators, but they no longer maintained that light acts via the oscillator driving sleep (Czeisler et al., 1986; Figure 2).

Ten years after our meeting in New York, Derk‐Jan Dijk conducted a milestone study in the laboratory of Charles Czeisler using the forced desynchrony paradigm to assess the contribution of homeostatic and circadian components to sleep regulation (Dijk & Czeisler, 1995). The essential tenets of the two‐process model were confirmed, and a quasi‐equal contribution of S and C to most variables with the exception of SWA was demonstrated. REM sleep was shown to be regulated by the circadian system and a sleep‐dependent disinhibition.^3^

A non‐linear interaction of the two processes was observed, because at times of high sleep pressure the circadian rhythm of waking vanishes (Dijk & Czeisler, 1995, figure 6). A similar observation was made in a recent omics study where high sleep pressure abolished the daily oscillation of the proteome, but not of the transcriptome (Noya et al., 2019).

## 4 TESTING THE MODEL AND RELATED DEVELOPMENTS

In the following I shall summarize the research projects between 1987 and 1997, which were devoted to testing various aspects of the model and gave rise to new developments in sleep science. Literature references are provided only for some key papers. For other references the reader is referred to Borbély and Achermann (1999), Achermann and Borbély, (2017), Borbély et al. (2016) and Borbély (2019). The collaboration with Anna Wirz‐Justice resulting in clinical applications of the model is discussed in Borbély et al. (2016).

A crucial first question was whether the homeostatic and circadian facets of sleep were separate processes. This prompted us to subject animals that had been rendered arrhythmic by lesioning the suprachiasmatic nucleus to sleep deprivation (Tobler et al.,^1983^). As the rise in SWS was still present, we concluded that an intact circadian pacemaker is not a prerequisite for sleep homeostasis.

Derk‐Jan Dijk, first as a doctoral student in Groningen and then as a postdoc in Zurich, performed a series of human studies between 1983 and 1996 to test the predictions of the model. The experimental protocols included the selective suppression of SWA, long sleep beginning in the evening, repeated partial sleep deprivation, recovery sleep in the morning, sleep extension and sleep in different age groups. Sleep largely conformed to the predictions of the model.

In 1983, Peter Achermann joined my group for a doctoral thesis and became one of our principal collaborators. With his diploma as an electrical engineer, he was optimally qualified to oversee signal analysis and to further elaborate the model. He incorporated the ultradian dynamics of SWA, and in a milestone paper performed a quantitative simulation of its time course for various protocols (Achermann et al., 1993).

The model had its origin in animal studies and, with the development of spectral analysis of the EEG, animal experiments were continued. In 1975, Irene Tobler, a biologist, joined my group as a doctoral student. Whereas initially she worked on the neurochemistry of sleep, from 1984 on she became involved in testing the two‐process model. Her fascination with zoology led her to conduct sleep studies in 17 different species over the years (Borbély, 2019, p.169). In milestone experiments, she showed that a sleep‐like rest‐homeostasis is present in two invertebrate animals, cockroach (Tobler, 1983; Tobler & Neuner‐Jehle 1992) and scorpion (Tobler & Stalder, 1988). These insights opened the invertebrate domain to sleep research where studies in Drosophila witnessed an explosive growth.

During the entire period we benefitted from the continuing contacts with Serge Daan, Domien Beersma and Rudi van den Hoofdakker in Groningen. A grant from the European Training Program in Brain and Behaviour Research enabled for our teams mutual 2‐day visits that strengthened the bond and friendship. Two students from Groningen came to Zurich for a part of their thesis work. Paul Franken performed long‐term studies in the rat to test an adapted version of the two‐process model, and Tom Deboer explored the relationship between sleep and torpor in the Djungarian hamster. After their theses, both continued their research in Zurich as postdoctoral scientists.

An important development started with the mapping of EEG spectra over different brain areas. Esther Werth and Peter Achermann showed that after sleep onset, SWA exhibited a gradient between the anterior and posterior brain regions that in the course of sleep gradually vanished (Werth et al., 1996, 1997). This hyperfrontality of SWA was clear evidence for regional differences in sleep regulation, and paved the way for the concept of local sleep. Because frontal parts of the cortex are involved in complex tasks, they may have an increased need for high‐intensity sleep.

To investigate directly whether the selective activation of brain areas is followed by increased regional sleep intensity, we applied a prolonged vibratory stimulus to one hand (Kattler et al., 1994). In the first part of the following sleep period an interhemispheric shift of SWA towards the contralateral somatosensory projection area was observed. This was the first direct demonstration of a regional, use‐dependent facet of sleep, a finding that was confirmed in the rat using unilateral vibrissae stimulation (Vyazovskiy et al., 2000).^4^

In conclusion, the two‐process model sparked a series of human and animal experiments that were designed to test its predictions. In addition, it extended the notion of sleep to the realm of invertebrates, and it revealed that sleep has also a local facet. The latter insight led to the exploration of concomitants of sleep in specific regions and structures of the brain.

## 5 OUTLOOK

The modelling approach is still a powerful technique to explore sleep regulation at different levels of brain organization and to promote studies for testing predictions. To some of the recent projects Peter Achermann contributed his expertise. Thus, Mathilde Guillaumin showed in mice that the dynamics of SWA as a marker of Process S differs between frontal and occipital cortical areas, but is resilient to extrinsic influences that affect the distribution of vigilance states (Guillaumin et al., 2018). Chris Thomas reported in a milestone experiment that Process S can be derived from cortical firing rate alone, demonstrating that sleep homeostasis can be modelled entirely at the local level (Thomas et al., 2020). This leads to the question of how multiple local events are temporally and spatially integrated to yield a global behaviour.

According to the findings of Lukas Krone, cortical neuronal networks are actively involved in both sleep regulation and sleep–wake state control, as experimental silencing of the pyramidal neurons results in a marked reduction of sleep time and impairs homeostasis without affecting the circadian regulation (Krone et al., 2021).

Impressive demonstrations of unravelling the two intertwining processes with the help of modelling are provided by recent research in Paul Franken's group. Thus, the continuous monitoring of the clock gene product period‐2 (PER2) by bioluminescence revealed that its damped harmonic oscillation is driven by a sleep–wake‐dependent force and a circadian peripheral force (Hoekstra et al., 2021). Not only sleep deprivation but also spontaneous periods of wakefulness affected the central and peripheral dynamics of this protein. Using brain temperature at high temporal resolution in a state‐of‐the art follow‐up of an early study (Franken et al., 1992), a model was developed that predicted with high accuracy state‐related and long‐term changes, and provided data on the relative contribution of homeostatic and circadian factors (Sela et al., 2021).

The term “homeostat” has been used to designate a hypothetical brain structure or neurochemical process underlying sleep homeostasis. Although various concomitants of the homeostatic process such as adenosine have been specified, a homeostat as such has not been defined (see Heller, 2021 for a lucid discussion). Still, one of the most influential theories is known under the name of synaptic homeostasis hypothesis (Tononi & Cirelli, 2003). The authors propose that neuronal circuits increase in synaptic strength during waking and weaken during sleep. In a recent review, they discuss the complexity of the various neurochemical processes that are involved, and conclude that it is still unclear why some synapses are more targeted than others (Cirelli & Tononi, 2021). Both circuit‐specific weakening and strengthening of synapses occur during sleep (Frank & Heller, 2018).

There is a tendency to focus explorations at the synaptic level on the sleep–wake‐dependent effects because they are more accessible to experimental manipulation than circadian effects. However, also the latter play an important role. Thus, the synaptic excitation–inhibition (E–I) balance of cortical neurons undergoes slow set‐point changes across the day, and is different at the light–dark and dark–light transition (Bridi et al., 2020). There are also indications from a human EEG study for a slow daily regulation of the E–I balance (Chellappa et al., 2016).

The notion of local sleep opened the field to search for sleep correlates at the level of neuronal circuits or even in single cells. Astrocytes are also involved in sleep homeostasis, and regulate sleep depth and sleep duration via separate pathways (Vaidyanathan et al., 2021). In Drosophila, astrocytes may act as sensors of sleep need (Blum et al. 2021). However, it is still an open question how events at the cellular level are transformed into a sleep drive that results in the manifestation of sleep.

The view that a central circadian pacemaker controls rhythmic variations in the entire organism has been expanded by the recognition of multiple clocks acting at different levels as parts of a circadian network (for review, see Koronowski & Sassone‐Corsi, 2021). The clock circuitry connects neurons and astrocytes that possess their own molecular clocks and oscillate in antiphase to each other. The coupling of cellular clocks results in a tissue clock, and the coupling of tissue clocks results in an organismal clock with feedback present at all levels. In view of the coordinated balance among tissues and organs, the authors consider circadian rhythms as a prime example of a homeostatic control.^5^ The sleep–wake cycle in conjunction with the fasting–feeding cycle are entrainment cues for peripheral oscillations, and are also important for rhythm setting at the organismic level. They are critical for the daily variation in energy metabolism.

In conclusion, the two‐process model is still valid and relevant, and derives its conceptual power from its ability to analyse processes at different levels of organization. Invariably, a history‐dependent factor complements an intrinsic rhythmic factor that reflects the 24‐hr cycle of the environment. On the cellular and molecular level, the two processes closely interact and at times cannot be strictly separated (Franken et al., 2013). However, on the macroscopic level the attributions are more clear‐cut and the analysis of their mode of interaction is more accessible.

From an evolutionary point of view the internalized 24‐hr rhythm is the guiding principle. Living organisms needed to adapt their behaviour and metabolism to the predictable alternation of light and dark, warm and cold. During the active phase of the daily cycle, the interaction with the environment and the acquirement of energy sources prevail, while the resting phase is devoted to internal housekeeping and the exploitation of energy. This allows a temporal compartmentalization of ergotropic catabolism and trophotropic anabolism. Sleep as enforced rest allowing a micro‐management in the minute‐to‐hours domain increases the flexibility for responding to challenges.

Dawn and dusk are crucial phases of the 24‐hr cycle representing the transition between day and night. As already mentioned, in an early sleep study I studied the role of evening/morning (E‐M) oscillators that are supposed to be coupled to dusk and dawn. Forty years later, Steven Brown, co‐director of the Chronobiology and Sleep Research lab in Zurich, showed with his team that rhythmic synaptic transcripts anticipate dusk and dawn (Noya et al., 2019). The transcripts anticipating the active period are involved in synaptic signalling, whereas those anticipating the rest period are concerned with metabolism. The subsequent “need‐dependent” local translation of the transcripts are linked to sleep and waking. Walter Rudolf Hess, director of the Zurich Physiology Department and Nobel Prize awardee, published in 1932 a milestone paper on the regulation of sleep whose function he saw in economy and repair (Hess, 1932). In anticipation of chronobiology, Hess distinguished two functional phases of the autonomic nervous system, an insight that remains fruitful and valid in the present omics era.

## CONFLICT OF INTEREST

The authors declare no conflict of interest.

## Data Availability

Not applicable.

## Endnotes

1 Daily variations of EEG amplitude in NREM sleep of the rat were previously reported by Rosenberg et al. (1976).

2 “The sleep process: circadian and homeostatic aspects.”

3 In 2008, I was awarded the Peter C. Farrell Prize in Sleep Medicine by the Harvard Medical School. The laudatio included the following: “This two‐process model of sleep regulation has enlightened successive waves of students and researchers, and prodded them to understand its dynamics and to delve into its biology. This vision has become a bedrock of our field.”

4 In a follow‐up study, Vlad Vyazovskiy showed that innate handedness in the rat is reflected by regional EEG asymmetry during sleep (Vyazovskiy & Tobler, 2008).

5 Circadian homeostasis refers to the coordinated balance of the system where homeostatic failure results in pathology. This is different from sleep homeostasis that acts to maintain sleep within a physiological range.
